# Consumer Preference, Quality, and Safety of Organic and Conventional Fresh Fruits, Vegetables, and Cereals

**DOI:** 10.3390/foods10010105

**Published:** 2021-01-06

**Authors:** S.M.E. Rahman, Mahmuda Akter Mele, Young-Tack Lee, Mohammad Zahirul Islam

**Affiliations:** 1 Department of Animal Science, Bangladesh Agricultural University, Mymensingh 2202, Bangladesh; ehsan_bau@yahoo.com or; 2 Department of Horticulture, Kangwon National University, Chuncheon 24341, Korea; mele@kangwon.ac.kr; 3 Department of Food Science and Biotechnology, Gachon University, Seongnam 13120, Korea; ytlee@gachon.ac.kr

**Keywords:** antioxidant, phenolic content, sugar content, vitamin C

## Abstract

Growing and purchasing demand for organic fresh produce is increasing rapidly. Consumers are aware of health, environmental safety, pesticide harmfulness, nutrients, bioactive compounds, and safe food. Many research works are available on organic and conventional fresh produce. As organic fresh produce growing and purchasing demand is increasing, it has become necessary to review the recent trends in quality, safety, and consumer preferences of organic and conventional fresh food products. A few reports have been compiled on organic and conventional fresh produce. Researchers have started working on organic and conventional fresh produce with the help of modern technology to improve nutritional and functional quality, safety, and consumer preferences. Nutritional and functional quality, safety, and consumer preferences depend on cultivation techniques, treatment, crop cultivar, and appearance of products. Therefore, it is necessary to compile the literature on organic and conventional fresh produce based on quality, safety, and consumer preferences.

## 1. Introduction

Organic and conventional fruit, vegetable, and cereal quality, safety, and preferences are becoming an issue for producers and consumers considered in the literature. Consumers demand quality and safety of fresh produce. Fresh produce quality reflects on taste, color, nutritional value, and microbial safety [1,2].

As it is grown in an organic manner, there undoubtedly are no synthetic pesticides and fertilizers, which can be harmful to human health. While synthetic pesticides are not authorized in many countries for organic farming, growers use manure and compost as organic fertilizer [3,4]. Growing and consumption trends are increasing daily. For example, there has been around 90% sales increase in North America and Europe [5]. The organic food industry is convincing the public that it is healthier, tastier, and better for the environment. Consumers prefer organic produce due to socioeconomics and attitudes to human health and the environment.

The conventional system typically uses synthetic pesticides and fertilizers, which can be harmful to human health if growers use them improperly [4,6]. For the careful application of synthetic pesticides and fertilizers, growers need proper knowledge about plant nutrients and food safety. Adequate application of fertilizers, pesticides, and manure can be beneficial for human health.

It is very difficult to compare organic and conventional foods. A valid comparison requires the same cultivar, proximity of the farms, similar growing media, same climate conditions, and a similar growing system. In this review paper, we tried to compare organic and conventional fresh produce according to quality, safety, and consumer preferences. Moreover, we focused on fresh produce growing materials in the organic and conventional systems. This article highlighted an overview of the recent works on organic and conventional fruit, vegetable, and cereal production systems, product quality, product safety, and consumer preferences.

## 2. Consumer Perception of Fresh Produce

Organic fresh produce markets are the fastest-growing in agriculture. In the year 2018, the organic food revenue in the USA was 40.56 billion euros, followed by Germany (10.91 billion euros) and France (9.14 billion euros), China (8.09 billion euros), Italy (3.48 billion euros), Canada (3.12 billion euros), Switzerland (2.66 billion euros), UK (2.54 billion euros), Sweden (2.30 billion euros) and Spain (1.90 billion euros) [7]. The growing and purchasing trends may vary from one country to another because of consumer demand. The per capita consumption of organic food in 2018 was the highest in both Denmark and Switzerland (312 euros), followed by Sweden (231 euros), Luxemburg (221 euros), Austria (205 euros), France (136 euros), Germany (132 euros), and the USA (124 euros) [7]. Consumers agreed to pay more for pesticide-free fresh produce because of perceptions of the negative health impact of synthetic pesticides [8]. Paying more for organic produce mainly depends on higher income, and the negative impact—on pesticides [8].

Safe consumption depends on personal and economic factors, sociocultural factors, and marketing factors [9]. These are age, gender, education, and marital status (personal factors); income and purchase intentions (economic factors); family size, status, and composition (sociocultural factors); price, advertising, and place (marketing factors). Besides, organic food consumption is influenced by the 4 P’s: product, price, place, and promotion.

Fresh and sustainable consumption, extrinsic attributes, health, sensory appeal, weight concern, and social status are used to consider organic food products. Furthermore, consumer motives, preferences, and attitudes can vary depending on the product category. Consumers are willing to pay additional 0.18 euro/kg organic apples in Italy [10], whereas in Denmark, it is 0.72 euro/kg [11]. Marketing strategy, product competition, social status, product satisfaction, and income may influence product purchases. Consumers like to buy organic vegetables at a higher price [12] because of safety. They also mentioned that purchasing organic food depends on attitudes, experiences, health status, knowledge, income, price, and sales. Organic food price is 10–40% higher in Denmark than the conventional food price [13] and 50–100% higher in Romania [14] due to consumers’ demand and high production cost. Organic fruits are safer because there are no pesticide residues. Appearance, freshness, nutritive value, and taste determine organic food preferences of consumers. Consumers prefer organic food due to its healthfulness and less environmental impact [15]. As a result, there is an increasing trend to consume organic foods instead of conventional foods.

## 3. Physicochemical Quality of Fresh Produce

### 3.1. Firmness

Firmness is an essential parameter for fresh produce. Conventional kiwifruits showed higher firmness than organic ones [16] (Table 1). There is a correlation between firmness and the B, Ca, Se, and Si content. These elements help to increase the cell wall thickness of cherry tomato fruits [17,18,19]. Guilherme et al. [20] reported that organically grown sweet peppers showed higher level of firmness than conventionally grown sweet peppers. They also mentioned that organically grown sweet peppers had the highest Ca content. Firmness mainly depends on the maturity of fruits because less mature fruits showed higher firmness than the fully mature ones [19]. If the organic growing system contains a high content of B, Ca, Se, and Si, it may also increase fresh produce’s firmness.

Moreover, smaller fruits showed higher firmness than bigger ones [21,22]. Although a high concentration of Ca decreases the fruit size in kiwifruit, it did not increase the firmness. The N:Ca ratio influences the firmness. For example, the lower the N:Ca ratio, the lower the apple firmness in the organic growing system, but the higher the N:Ca ratio, the higher the firmness in the conventional growing system [21,23], because Ca increases the cell wall thickness. Firmness depends on the cultivation system, nutrient concentration, and cultivar. The conventionally grown blueberries performed better in firmness than the organic production system [24] due to the increased level of mineral content in the conventional system. Reganold et al. [25] mentioned that there were no significant differences between the organic and the conventional systems for strawberries.

### 3.2. Organic Acid Content

Less ripe fruits have enriched titratable acidity, for example, cherry tomato fruits, because during the ripening time, acid converts to sugar [1,6,17,26]. Organic apples, strawberries (Diamante), pears, and beetroots showed higher titratable acidity compared with the conventionally grown ones [25,27,28] (Table 2). It may happen due to less effect of nutrient concentrations in the conventional system. Conventionally grown strawberries (Lanai and San Juan), celery, tomatoes, and lettuce showed increased titratable acidity, whereas the decreased level of titratable acidity was showed in organic ones [4,25,28,29]. There was no difference between the organic and the conventionally grown apples and carrots [23,28]. Conventionally grown sweet peppers showed higher titratable acidity than the organic ones [20].

### 3.3. Minerals

Mineral concentration is an important factor for conventional cultivation. Faba beans showed the lowest Ca content among the crops [30] (Table 3) and it may happen due to low amount of Ca applied during growing. The organic plums showed higher P, K, Ca, Mg, Zn content and lower Na, Fe, Cu content compared with the conventionally grown ones [31]. Apple fruits that grew in an organic manner showed higher K, Ca, Mg, Na, Mn content than the conventionally grown ones. However, the conventionally grown apple fruits exhibited more Fe, Cu, B, Zn compared with the organic ones [27]. Uckoo et al. [32] reported that organic lemons showed the highest P content, whereas the conventionally grown ones showed the lowest. They also reported that the conventional lemons showed a higher K, Ca, Mg, Na, Zn, Fe content compared with the organic ones. This variation may happen due to the high nutrient concentration applied in the conventional system. High Ca content may increase fresh produce firmness [17]. The organic corn grains were rich in K, P, Mg, Fe, Zn, and the conventional corn grain exhibited high S and Mn content [33]. Organic lettuce, peppers, and tomatoes are rich in Cr, Cu, Fe, K, Mg, or Na. The higher the level of Na content, the higher the sugar content [18] and bioactive compounds [34] in fresh produce may be. The conventional lettuce, peppers, and tomatoes are rich in Mn, Zn [35].

Conventional strawberry fruits showed higher P, K, Ca, Mg, B, and Zn content than the organic ones [25]. More conventional crops showed a higher N content than the organic ones [36] because in the conventional systems, N-based fertilizers are usually used more frequently. There is a positive correlation between conventional and N-based fertilizers. For example, organic tomatoes and strawberries have an increased K content, while among the conventionally grown produce, lettuce, potatoes, melons, and watermelons have an increased K content. Moreover, P showed a similar trend in organic tomatoes and strawberries, but potatoes and lettuce showed the opposite. Siderer et al. [37] reported that conventionally grown vegetables have more nutrients than the organic ones.

## 4. Sensory Quality of Fresh Produce

Sensory qualities of organic and conventional fresh produce may differ across crops and the findings are inconsistent [38] (Table 4). They also mentioned that even if organic foods are not superior in sensory qualities, they address safety and environmental considerations. Organic growing methods adversely affect sensory properties because they depend on the fertilizer type, not climate, soil, or other factors [23,38,39]. Sensory evaluations are performed in 3 ways: (a) discrimination/differences; (b) descriptive assessment (juiciness, sweetness, tartness, off-flavor, firmness, color, acidity, bitterness, crunchiness, and taste); and (c) acceptability/preferences/liking (appearance).

The organic lettuce maintained excellent sensorial characteristics as they developed a higher carbon dioxide concentration compared with the conventionally grown ones. Visual quality and shelf life are essential parameters for consumers because they influence the choice, selection, and purchase intentions. The quality of fruit depends on visual characteristics (size and color) [22,40], postharvest life or visual appearance, and consumer acceptability [41]. Organic fruits and vegetables performed better in visual quality and shelf life compared with the conventionally grown ones. For example, organic lettuce exhibited a longer shelf life than the conventionally grown ones [42].

The color of organically and conventionally produced fruits and vegetables differs significantly. Color is directly associated with consumer acceptability. Poor color development leads to loss of the fresh produce’s market value [21]. Among strawberries, the organic ones exhibited a darker red than the conventionally grown ones, and the shelf life was also longer [25]. Anthocyanin and lycopene prolong the shelf life of fruits and vegetables. Reganold et al. [25] also mentioned that organic strawberries have higher resistance power and better shelf life than the conventionally grown ones because the mineral balance affects physiology and firmness.

## 5. Organic and Conventional Methods of Growing Fresh Produce

**Figure 1:**
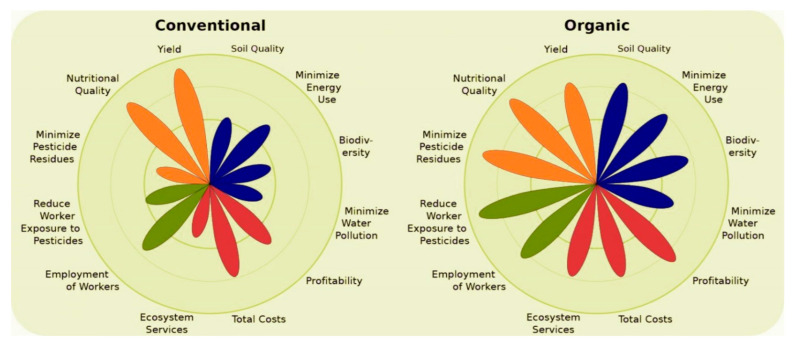
Difference between conventional and organic farming [47].

Organic fresh produce demand is increasing [7]. We need to confirm that organic fresh produce is rich in nutritional quality for human health. The improved nutritional value mainly depends on the cultural system, treatment, growing position, and environment. In the past two decades, many different research works have been compiled on organic and conventional systems [3,4,29,43,44,45,46]. According to Reganold and Wachter, [47] conventional cultivation showed higher yield and higher total cost. The impact of organic and conventional methods on the quality of fresh produce is presented in Figure 1.

The quality of fresh produce grown using organic and conventional methods is shown in Table 5. Among blueberries, the content of fructose, glucose, citric acid, malic acid, anthocyanin, total phenols, and flavonoids was higher in the organic ones than in the conventional produce [43]. Jin et al. [44] reported that organically grown strawberries contained higher glutathione, ascorbic acid, total anthocyanin, total phenolic acids, and had higher antioxidant activity compared to the conventionally grown ones. Organic tomatoes contained high sugar, phenols, flavonoids, whereas the conventionally grown ones had high acidity and high content of total polyphenols [29]. Organic bell peppers showed high dry matter, vitamin C, total carotenoids, total phenolic acids, quercetin, and kaempferol and conventional bell peppers showed high antheraxanthin, lutein, total flavonoids, myricetin, and luteolin [45]. In organic beetroots, dry matter, sugar, and vitamin C were increased [3], while antioxidant activity and ascorbic acid content were increased in lettuce [4]. Conventional beetroots showed high total polyphenols, flavonoids, and quercetin [3], while conventional lettuce showed high total soluble solids content, titratable acidity, total phenolic and total chlorophyll content [4]. Therefore, quality variation depends on the treatment, crops, and cultivation practice, but it is not possible to assign positive effects only to organic farming [3,4,29,43,44,45].

## 6. Nutritional Quality of Fresh Produce

In this section, soluble solids, sugar content, dry matter content, and dietary fiber are discussed. Increase and decrease in nutritional quality may depend on growing conditions and environmental factors.

### 6.1. Soluble Solids Content

The soluble solids content is an important parameter for consumer preferences. A higher soluble solids content is often associated with better taste [46]. The seawater treatment may convert organic acids to soluble sugar. In organic apples and strawberries (Diamante and San Juan), the soluble solids content increased [25,27] (Table 6). In the conventional cultivation system, the soluble solids content also increased in strawberries (Lanai), tomatoes, beets, and lettuce [3,4,6,20,25,27]. Some discovered that organic tomatoes, oranges, kiwifruits, lemons, and mandarins are rich in soluble solids [48,49]. Benge et al. [50] mentioned that conventional kiwifruits showed higher soluble solids than the organic ones, resulting in maturity. They mentioned that organic kiwifruits were probably harvested less mature. The increase and decrease in the soluble solids content may be influenced by cultivar, cultivation practices, treatment, nutritional concentration, and maturity stages of harvesting fresh produce.

### 6.2. Sugar Content

Organic pears, blackcurrants, beetroots, celery, kiwifruits, and tomatoes showed higher sugar content compared with the conventionally grown ones [3,28,29] (Table 6). Organic fruits contain less sugar [31] due to less Na, because Na treatment enhances the sugar content [18]. In contrast, the increased sugar content was found in conventional strawberries, apples, carrots, and cabbages [25,28,51]. Guilherme et al. [20] found that conventional and red peppers showed higher total soluble solids than the organic and green ones. The increase and decrease in sugar content may be influenced by crops, cultivars, treatment, and maturity of fresh produce.

### 6.3. Dry Matter Content

Organic melons, tomatoes, potatoes, beets, and watermelons showed increased dry matter [28] and conventional strawberries, wheat, barley, faba beans, and lettuce exhibited high dry matter [30,36] (Table 7). Organic cabbages, red beets, peppers, tomatoes, and potatoes showed high dry matter content [20,45,52]. Moreover, the perilla and cabbage total dry matter content is higher in organic produce than in the conventionally grown one [51,53]. The dry matter content increases and decreases may happen due to water absorption by the plant. There was no difference in the water content of organic and conventional plums [31].

Dry matter content increases the flesh product of organic lettuce and, as a result, produce shows less decay and decomposition with increased storage life [42]. Yu et al. [33] reported that dry matter content accumulated by photosynthesis is almost 7–20% higher in organic crops than in the conventionally grown ones. However, conventional potato tubers had more dry matter than organic tubers [54]. There is no difference in the dry matter content of beetroots, cabbages, and carrots [3].

### 6.4. Dietary Fiber

Conventional plums, grapes, and wheat performed better in total dietary fiber and soluble fiber than the organic ones [31,55] (Table 7). In insoluble fiber, no differences between organic and conventional plum cultivation systems were found. The total dietary fiber content of the conventional Bordô grape flour and pumpkins was higher than of the organic ones [55,56]. Fruits and vegetables are a good source of dietary fiber. They are rich in bioactive compounds, which reduce the bioavailability of fat in the human diet. The dietary fiber might lower the bioavailability of carotenoids. Organically grown *Talinum triangulare* and pumpkins contained more dietary fiber than the conventionally grown ones [56,57]. The most important is the growth/differentiation balance (GDB) hypothesis. Growth denotes the production of roots, stems, and leaves or cell division and elongation; differentiation indicates the enhancement of the structure or function of the existing cells [58]. In the case of the GDB hypothesis, growth and differentiation are necessary for primary and secondary metabolism [59]. Secondary metabolism gives a distinct aroma in grapes [60]. The conventionally grown sweet peppers showed higher fiber content than the organic ones at both the green and the red maturity stages [20].

## 7. Bioactive Compounds of Fresh Produce

In this section, the content of total phenolic acids, vitamin C, β-carotene, anthocyanin, flavonoids, lycopene, and antioxidants of organically and conventionally grownn fresh produce was discussed.

### 7.1. Total Phenolic Content

Phenolic acids are antioxidants and secondary metabolites that have a functional role against cardiovascular diseases, neurological diseases, and cancer. Phenolic content variation mainly depends on the cultivar, growing condition, and treatment. For instance, Feteasca regală, Muscat Ottone, Napoca, Chasselas doré, and Muscat Hamburg cultivars of organic grapefruit skin showed higher polyphenol content than Aromat de Iaşi, Traminer Roz, Riesling Italian, and Timpuriu de Cluj cultivars of conventional grapefruit skin [61]. Some researchers found that organic produce showed a lower level of polyphenols due to low N compared with the conventionally grown one [36].

Organic broccoli, blueberries, grapefruits, peppers, plums, potatoes, strawberries, and tomatoes are rich in phenolic compounds [6,43,44,45] (Table 8). Moreover, organic grapes or fruits and vegetables showed higher phenols than the conventionally grown ones [62]. High phenolic content of organic fruits and vegetables mainly depended on the pathogenic pressure that influenced the phenolic biosynthesis because of their defense mechanism. Some have argued that conventional strawberries and oranges exhibited high phenolic content [41].

### 7.2. Vitamin C Content

Vitamin C is an antioxidant and it has an influential role in scurvy. It is effective against microorganisms. Organic strawberries, beetroots, and lettuce showed higher vitamin C content than the conventionally grown ones [3,4,41] (Table 8). Organic blueberries [63], citruses [48], plums [64], strawberries [49], tomatoes [48], and other plant foods [65] contained higher amounts of vitamin C than the conventionally grown ones.

According to Barrett et al. [66], higher and lower vitamin C content in organic and conventional tomatoes were found. It may happen due to the maturity stage, because the ripening progress reduces the vitamin C content [17]. However, Hunter et al. [65] reported that organic plants showed 10.4% higher amounts of vitamin C than the conventionally grown ones. The variation may happen due to cultivar and growing conditions. Organic produce showed more vitamin C as it presented lower amounts of nitrogen than the conventional crops [36]. In contrast, conventional potatoes exhibited higher amounts of vitamin C than the organic ones [67]. Besides, some research mentioned that there are no differences in the vitamin C content of organic and conventional green beans, tomatoes, capsicum, and silver beets [68].

### 7.3. β-Carotene Content

Carotenoids are natural pigments that are contained in fruits and vegetables. Carotenoids have a functional role as an anti-cancer, anti-oxidation, eyesight, osteoporosis, and skin aging prevention agent. Organic peppers and tomatoes exhibited higher β-carotene content than the conventionally grown ones [29,48,52] (Table 9). Some researchers also mentioned that β-carotene content is higher in organic broccoli, citruses, strawberries, cabbages, persimmons, and peppers than in the conventionally grown ones [33,49,51,69,70].

In contrast, the level of carotenoids is often lower in organic passion fruit compared with the conventionally grown ones [71]. Increased or decreased β-carotene content depends mainly on the cultivar and growing conditions. For example, β-carotene content is increased in Aromat de Iaşi, Riesling Italian, Napoca, Chasselas doré, and Muscat Hamburg cultivars of organic grapefruit skin. Such cultivars as Traminer Roz, Feteasca regală, Muscat Ottone, and Timpuriu de Cluj showed a higher β-carotene content in conventional grapefruit skin than in the organic one [61].

### 7.4. Anthocyanin

Anthocyanin is a bioactive compound, which is found in colored fruits. Anthocyanin is a plant pigment, which is synthesized in epidermal layers. Organically cultured strawberries exhibited higher anthocyanin content than the conventionally grown ones [25,41] (Table 9); this may happen due to rapid ripening. Some further mentioned that anthocyanin content is higher in organic blueberries, grapes, and plums than in the conventionally grown ones [33,43,62,64].

In the case of blueberries, there is no specific trend. Some cultivars showed high, and some showed low anthocyanin content. For instance, cultivar “Powder blue” showed high anthocyanin content in the organic system and cultivars “Climax” and “Woodward” showed high anthocyanin content in the conventional cultivation system [72]. So, both the cultivar and the growing system influenced the anthocyanin content of fruits.

### 7.5. Flavonoids

Levels of the flavonoid content were more increased in conventional apples, beetroots, and cabbages than in the organic ones [3,27,51] (Table 10). However, tomatoes showed the opposite result. Tomatoes showed higher flavonoid contents in organic compared with the conventionally grown ones [29]. Yu et al. [33] mentioned that the isoflavone content was higher in organic foods. Similarly, organic broccoli contained a higher flavonoid content [73], which may happen due to maturity.

Grapefruit flavanone content was described by Chebrolu et al. [74] during harvest time and storage. They mentioned that in organic grapefruits, narirutin, neohesperidin, and didymin were high at the harvest time in the first experiment conducted in November. In contrast, conventional grapefruits showed higher naringin and poncirin at the harvest time in the first experiment. Flavanones (narirutin, neohesperidin, naringin, poncirin, and didymin) were higher in the conventional grapefruits grown in February [74]. During the storage period, all flavanones were increased due to ripening. A similar trend is also found in conventional blueberries regarding caffeic acid, chlorogenic acid, p-coumaric acid, and quercetin [72]. Conventionally grown Maltese demi-sanguine blood oranges showed a higher gallic acid, sinapic acid, caffeic acid, p-hydroxybenzoic acid, p-coumaric acid, ferulic acid, vanillic acid, narirutin, naringin, and narirutin content compared with the organic ones. However, hesperidin showed the opposite [75].

### 7.6. Lycopene

Lycopene is contained in ripening fruits with light-red to red colors. For instance, red cherry tomatoes showed higher lycopene content compared with the light-red ones [1,6,26]. Organic persimmons and tomatoes (Merkury, Akord, Rumba, Redondo) showed higher lycopene content than the conventionally grown ones [6,29,69] (Table 10). In contrast, conventional tomatoes (Picolino, Conchita) and passion fruit exhibited higher lycopene content than the organic ones [29,48,71]. So, lycopene is influenced by the cultivar, stage of maturity, and growing system.

### 7.7. Antioxidant Activity

Antioxidant activity in organic strawberries, pears, carrots, blackcurrants, beetroots, eggplants, peppers, spinach, plums, and celery was higher than in the conventionally grown ones [25,28,64,74,76] (Table 11). However, apples and blueberries did not show a similar trend. In apples and blueberries, some cultivars showed higher and some showed lower antioxidant activity in organic and conventional systems. For example, organic apples (Starking Delicious, Golden Delicious, Jona Gold) and blueberries (Powder blue) had increased antioxidants. In contrast, some conventional apples (Granny Smith, Royal Gala), blueberries (Climax, Woodward), and tomatoes had increased antioxidants [43,48,72,77].

It was further discovered that organic vegetables showed higher antioxidant content due to lower N availability [78]. Conventional blueberry fruits, blueberry seeds, and skin extracts had enriched antioxidant capacity (hydrophilic oxygen radical absorbing capacity test) [63]. Organic microgreen extract exhibited high antioxidant concentrations (2,2′-azino-bis (3-ethylbenzothiazoline-6-sulfonic acid and 2,2-diphenyl-1-picryl-hydrazyl-hydrate assay) [34,79]. Antioxidant content was influenced by the cultivar. Grapefruit skin cultivars (Aromat de Iaşi, Traminer Roz, Riesling Italian, Muscat Ottonel, Napoca, Chasselas doré, and Muscat Hamburg) have a higher antioxidant content when grown in an organic manner [61]. Moreover, other cultivars (Feteasca regală and Timpuriu de Cluj) have high antioxidant content in the conventional grapefruit skin.

## 8. Food Safety of Fresh Produce

Microbial contamination risk may increase in organic farming due to *E. coli*, mycotoxins, and parasites because of the limitation of pesticides (fungicides, insecticides, and herbicides) in organic farming. It is necessary to find the proper interval between applying manure and harvesting fresh produce to minimize the risk of microbial contamination (*Salmonella enterica*, *S. typhimurium*, and *E. coli* 0157:H7). Fungal toxins are also a threat to human health. For example, in the storage period, bacterial and fungal incidence can increase in cherry tomato fruits and leafy vegetables due to internal functional activity resulting from quality deterioration [2,26]. Bourn and Prescott [38] mentioned that they did not find any differences in organic and conventional foods in the aspects of microbiological contamination. Aerobic mesophilic bacteria, yeasts, molds, and coliforms are higher in conventional than organic vegetables [80]. Organic farming practices with animal manure can increase the risk of contamination by pathogenic microorganisms which may pose health risks. A number of scientific studies on the microbial quality of organic and conventional fresh produce has been conducted; their results are contradictory. While some have a greater microbial count in fresh organic produce, other studies do not [81]. Fruits and vegetables are usually contaminated by soil, water, manure, and wild animals. In this regard, good hygiene practices should be implemented to prevent contamination [82].

**Figure 2:**
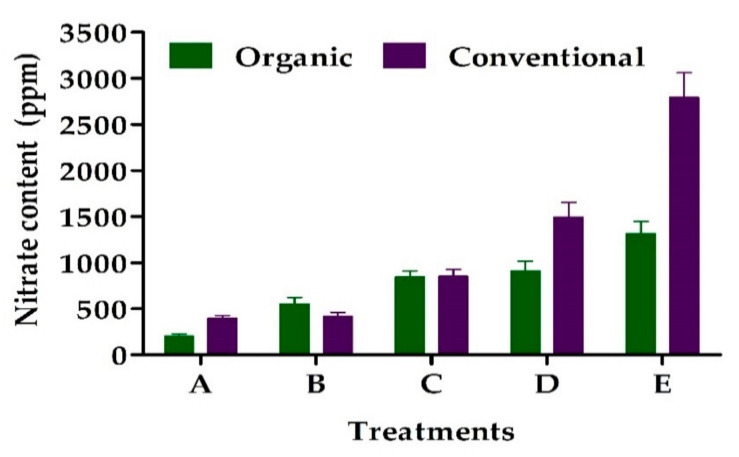
Nitrate (NO_3_^−^) content of organic and conventional broccoli (A), cabbages (B), lettuce (C), celery (D), and spinach (E) in the USA. Source—de González et al. [84].

Nitrates are naturally present in plant products and are considered a matter of concern for health due to their easy conversion to nitrites and their reactivity (Figure 2). Nitrites oxidize hemoglobin, which causes acute intoxication and cancer [33]. The maximum daily intakes of nitrates and nitrites recommended by the European Food Safety Authority are 3.7 and 0.07 mg/kg body weight, respectively [83]. There was increased nitrate content in organic carrots and strawberries, conventional watermelons, tomatoes, carrots, strawberries, potatoes, lettuce, and melons [36]. Moreover, conventional green vegetables had a higher nitrate content than the organic ones [68]. However, some reported the opposite result for citruses and strawberries [49]. Nitrate accumulation in fresh produce is mainly due to nitrogen, and low nitrogen can reduce the nitrate content. Organic fruits and vegetables had fewer nitrates than conventional crops as they had lower amounts of nitrogen [21,33,36]. Therefore, the nitrate availability in plants depended on the climate, cultivar, and crop rotation, date of harvesting, date of planting, diseases of the plant, growing location, irrigation, and soil type [36,78].

The ban on toxic chemicals like fungicides, insecticides, and herbicides in organic agriculture ensures the protection of workers’ health. Organic foods of plant origin did not contain any pesticide residues [38]. Organic foods contained minimal levels of toxic heavy metals and cadmium, higher antioxidant and lower pesticide residues than the conventionally grown ones.

Aflatoxins occur in nut trees, dried fruits, cereals, and coffee [85]. They have severe implications for human health because they work as a human carcinogen [21]. Gourama [86] did not find any aflatoxin content in organic almonds, but found it in conventionally grown foods. The *Aspergillus* group produces aflatoxins, mainly, *Aspergillus flavus* produces aflatoxin B_1_ and B_2_ and *A. parasiticus* produces aflatoxins B_1_, B_2_, G_1_, and G_2_ [86]. Organic crops contain high carbohydrates due to low nitrogen application; as a result, organic foods are more susceptible to the consequences of fungal infections and produce more aflatoxin B_1_.

## 9. Conclusions and Future Perspective

This paper discusses consumer preference, quality, and safety of organic and conventional fresh produce. The studies evaluated included information on consumer perception, physicochemical quality, sensory quality, nutritional quality, bioactive compounds, and food safety. Most of the differences detected between organic and conventional fresh produce could be strongly linked to differences in crops, fertilizers, environment, and pest management. According to the literature reviewed, consumers have a higher perception of organic fresh produce than of the conventional produce and are willing to pay additional money. They prefer organic fresh produce, which is free from synthetic fertilizers, fungicides, insecticides, and herbicides. Physicochemical quality, mainly, firmness was high in organic apples, strawberries, kiwifruits, and sweet peppers. Conventionally grown celery, lettuce, tomatoes, and sweet peppers contained high levels of organic acids. Organic growing methods adversely affect sensory properties because they depend on the fertilizer type, not climate, soil, or other factors. Conventionally grown grapes, sweet peppers showed abundant dietary fiber, whereas in organically grown fruits, the increased soluble solids (apples and beetroots) and sugar content (beetroots) depends on growing conditions. Organic fresh produce has high phenolic content. This may happen due to the endogenous phenolic-enriching plant defense mechanisms in the absence of synthetic fertilizers and pesticides commonly used in the conventional growing system [21,87]. The zero-use of pesticides is linked to higher antioxidant levels in organic fresh produce than in the conventionally grown one [21]. Conventionally grown vegetables have a higher nitrate content than the organic ones. Proper interval between applying manure and harvesting fresh produce is required to minimize the risk of microbial contamination. Besides, more studies are needed to standardize production, quality, safety, and consumer preferences regarding organic and conventional fresh produce to fulfill consumer demand.

## Figures and Tables

**Table 1:** The firmness content of organic and conventional fresh produce.

Fresh Produce	Firmness		References
	Organic	Conventional	
Apples	Full ripeness: 78–82 (N)	Full ripeness: 72–78 (N)	[23]
Strawberries	Full ripeness: 4.36 (N)	Full ripeness: 4.17 (N)	[25]
Sweet peppers	Green: 12.1, red: 9.2 (N)	Green: 7.9, red: 7.9 (N)	[20]
Blueberries	Full ripeness: 149 (G/mm)	Full ripeness: 199 (G/mm)	[24]

**Table 2:** The organic acid content of organic and conventional fresh produce.

Fresh Produce	Organic Acid Content		References
	Organic	Conventional	
Apples	Starking Delicious: 0.31 (g malic acid/100 mL)	Starking Delicious: 0.30 (g malic acid/100 mL)	[27]
Strawberries	Diamante: 9.16, Lanai: 7.18, and San Juan: 6.97 (mg citric acid/g Fresh weight)	Diamante: 7.52, Lanai: 7.51 and San Juan: 7.79 (mg citric acid/g Fresh weight)	[25]
Apples, pears, carrots, blackcurrants, beetroots, and celery	Red Boskoop (apples): 0.93, Bartlett (pears): 0.42, Tiben (blackcurrants): 4.00, Perfection (carrots): 0.09, Czerwona Kula (beetroots): 0.12, and Jabłkowy (celery): 0.46 (g malic acid/100 g)	Red Boskoop (apples): 0.71, Bartlett (pears): 0.23, Tiben (blackcurrants): 3.00, Perfection (carrots): 0.09, Czerwona Kula (beetroots): 0.10, and Jabłkowy (celery): 0.61 (g malic acid/100 g)	[28]
Tomatoes	Merkury, Akord, Rumba: 8.54 (g) Picolino and Conchita: 7.72 (g)	Merkury, Akord, Rumba: 9.56 (g) Picolino and Conchita: 9.04 (g)	[29]
Lettuce	Caipira: 0.0047–0.0059 (g citric acid/kg)	Caipira: 0.0067 (g citric acid/kg)	[4]
Sweet peppers	Green: 63, red: 162 (g citric acid/100 g)	Green: 67, red: 171 (g citric acid/100 g)	[20]

**Table 3:** The mineral content of organic and conventional produce.

Mineral Content															
		K	Mg	P	S	Ca	Fe	Mn	B	Zn	Cu	Mo	Ba	Sr	Na
	GC	(%)					(mg/kg)								
Wheat grains	ORG	0.41	0.11	0.34	0.11	311	33	19	-	19	3.7	0.5	2.8	2.5	37
	CON	0.43	0.10	0.32	0.12	322	38	28	-	19	3.8	0.3	3.0	2.6	27
Barley grains	ORG	0.47	0.10	0.34	0.11	378	37	9	-	21	3.5	0.3	1.8	2.0	60
	CON	0.43	0.10	0.31	0.12	382	37	13	-	20	3.7	0.2	1.6	2.0	53
Faba beans	ORG	1.47	0.18	0.64	0.21	0.17	64	21	7.3	53	15.5	1.4	0.5	3.4	129
	CON	1.50	0.17	0.64	0.22	0.17	64	23	7.7	49	14.4	1.1	0.7	3.7	84
Potatoes	ORG	2.28	0.11	0.24	0.16	180	21	6	4.5	12	5.3	0.4	0.3	0.8	69
	CON	2.08	0.11	0.21	0.15	206	20	6	4.6	10	4.3	0.2	0.3	0.9	49

**Note:** K-S = % and Ca-Na = mg/kg. GC—growing condition, ORG—organic, CON—conventional, K—potassium, Mg—magnesium, P—phosphorus, S—sulfur, Ca—calcium, Fe—Iron; Mn—manganese, B—boron, Zn—zinc, Cu—copper, Mo—molybdenum, Ba—barium, Sr—strontium, and Na—sodium. Source—Laursen et al. [30].

**Table 4:** Sensory quality of organic and conventional fresh produce based on a 9-point scale.

Fresh Produce	Consumer Sensory Quality		References
	Organic	Conventional	
Apples	Overall: 6.2–6.8; texture: 6.5–6.8; flavor: 5.9–6.6; firmness: 6.6–6.8; sweetness: 5.0–5.6; tartness: 4.7–4.9.	Overall: 6.5–6.7; texture: 6.3–6.8; flavor: 6.2–6.5; firmness: 6.1–6.4; sweetness: 5.8–5.8; tartness: 4.3–4.5.	[23]
Lettuce	Overall liking: 5; flavor intensity: 4.5; bitterness intensity: 4.4	Overall liking: 5.4; flavor intensity: 4.2; bitterness intensity: 3.9	[39]
Mustard greens	Overall liking: 2.7; flavor intensity: 6.6; bitterness intensity: 5.2	Overall liking: 3; flavor intensity: 6.5; bitterness intensity: 5.2	[39]
Tomatoes	Overall liking: 7; flavor intensity: 4.6; other attributes: 4.7	Overall liking: 7; flavor intensity: 5; other attributes: 5	[39]
Cucumbers	Overall liking: 7.1; flavor intensity: 4.1; other attributes: 2.3	Overall liking: 7; flavor intensity: 4; other attributes: 2.2	[39]

**Table 5:** The impact of organic and conventional methods on the quality of fresh produce.

No.	Cultural Methods	Fresh Produce	Treatment	Quality	References
1	Organic and conventional	Blueberries	Organic: cover crops, peat, compost, fish meal, humus, and manure and organic herbicides (crop oils, vinegar, and soaps) Conventional: NPK fertilizers, pesticides, herbicides, insecticides, and fungicides	Organic: fructose, glucose, citric acid, malic acid, anthocyanin, total phenolic acids, and flavonoids are high. Conventional: fructose, glucose, citric acid, malic acid, anthocyanin, total phenolic acids, and flavonoids are low.	[43]
2	Organic and conventional	Strawberries	Organic: horse manure, granite dust, no herbicides or insecticides, NPK from steamed bone meal, feather meal, soybean meal, langbeinite, and compost. Conventional: NPK fertilizers, fungicides Switch and Captec, insecticides Lorsban and Brigade, herbicides Stinge and Herbimax.	Organic: glutathione, ascorbic acid, total anthocyanin, total phenolic acids, and antioxidant activity are high. Conventional: glutathione, ascorbic acid, total anthocyanins, total phenolic acids, and antioxidant activity are low.	[44]
3	Organic and conventional	Tomatoes	Organic: green manure, 60% compost; biohumus, cow manure Conventional: NPKMgS fertilizers	Organic: sugar, phenol, flavonoids are high. Conventional: acidity and total polyphenols are high.	[29]
4	Organic and conventional	Bell peppers	Organic: green manure, compost, cow manure, organic protection. Conventional: NPKS fertilizers, synthetic protection.	Organic: dry matter, vitamin C, total carotenoids, total phenolic acids, quercetin, and kaempferol are high. Conventional: antheraxanthin, lutein, total flavonoids, myricetin, and luteolin are high.	[45]
5	Organic and conventional	Beetroots	Organic: N (low and high) from compost and manure, no pesticides. Conventional: N (low) with pesticides.	Organic: dry matter, sugar, and vitamin C are high. Conventional: total polyphenols, flavonoids, and quercetin are high.	[3]
6	Organic and conventional	Lettuce	Organic: Altavita, Altaverde, cow and chicken manure, no pesticides. Conventional: NPK with deep irrigation.	Organic: antioxidant activity and ascorbic acid content are high. Conventional: total soluble solids content, titratable acidity, total phenolic content, and total chlorophyll content are high.	[4]

Note: No. 6 refers to the greenhouse soil and the rest of them refer to field soil.

**Table 6:** Soluble solids and sugar content of organic and conventional fresh produce.

Fresh Produce	Soluble Solids Content		References
	Organic	Conventional	
Apples	Starking Delicious: 12.66 (Brix)	Starking Delicious: 12.40 (Brix)	[27]
Strawberries	Diamante: 8.97, Lanai: 8.98, and San Juan: 8.96 (Brix)	Diamante: 7.68, Lanai: 9.52, and San Juan:8.71 (Brix)	[25]
Tomatoes	Redondo: 4.38 (Brix)	Redondo: 4.46 (Brix)	[6]
Beetroots	Libero: 6.0–7.4 (g/kg FW)	Libero: 6.1–6.3 (g/kg FW)	[3]
Lettuce	Caipira: 28–29 (g/kg)	Caipira: 36 (g/kg)	[4]
Sweet peppers	Green: 3.8, red: 5.8 (Brix)	Green: 4.4, red: 7.6 (Brix)	[20]
**Sugar content**			
Apples, pears, carrots, blackcurrants, beetroots, and celery	Red Boskoop (apples): 7.9, Bartlett (pears): 6.9, Tiben (blackcurrants): 7.7, Perfection (carrots): 5.6, Czerwona Kula (beetroots): 8.4, and Jabłkowy (celery): 1.3 (%)	Red Boskoop (apples): 9, Bartlett (pears): 6.7, Tiben (blackcurrants): 7.5, Perfection (carrots): 7.1, Czerwona Kula (beetroots): 5.9, and Jabłkowy (celery): 0.6(%)	[28]
Tomatoes	Merkury, Akord, Rumba: 85.22 (g) Picolino and Conchita: 88.93 (g)	Merkury, Akord, Rumba: 83.48 (g) Picolino and Conchita: 78.12 (g)	[29]
Beetroots	Libero: 131.1–142.6 (g/kg FW)	Libero: 125.8–129.6 (g/kg FW)	[3]
Cabbages	Sufama F1: 6.59 (g/100 g FW)	Sufama F1: 6.63 (g/100 g FW)	[51]

**Table 7:** Dry matter and dietary fiber content of organic and conventional fresh produce.

Fresh Produce	Dry Matter Content		References
	Organic	Conventional	
Wheat, barley, faba beans, potatoes	Wheat: 89.4, barley: 89.5, faba beans: 88.1, potatoes: 19.2 (%)	Wheat: 89.6, barley: 90.3, faba beans: 87.9, potatoes: 20.5 (%)	[30]
Apples, pears, carrots, blackcurrants, beetroots, and celery	Red Boskoop (apples): 12.4, Bartlett (pears): 12, Tiben (blackcurrants): 15.2, Perfection (carrots): 9.7, Czerwona Kula (beetroots): 12.2, and Jabłkowy (celery): 10.4 (%)	Red Boskoop (apples): 13.4, Bartlett (pears): 11.2, Tiben (blackcurrants): 12.6, Perfection (carrots): 10.4, Czerwona Kula (beetroots): 8.3, and Jabłkowy (celery): 8.9 (%)	[28]
Bell peppers	Roberta: 82.2 (g/kg)	Spartacus: 75.9 and Berceo: 78.4 (g/kg)	[45]
Beetroots	Libero: 163.7–182.4 (g/kg FW)	Libero: 163.7–182.4 (g/kg FW)	[3]
Cabbages	Sufama F1: 9.22 (g/100 g FW)	Sufama F1: 8.81 (g/100 g FW)	[51]
Sweet peppers	Green: 6.38, red: 5.87 (%)	Green: 4.98, red: 5.03 (%)	[20]
**Dietary fiber content**			
Grapes	Flour: 62.70 (%)	Flour: 69.70 (%)	[55]
Pumpkins	2.68 (%)	2.47 (%)	[56]
*Talinum triangulare*	42–79 (g/100 g Deionized water, DW)	40–73 (g/100 g DW)	[57]
Sweet peppers	Green: 11.4, red: 9.23 (%)	Green: 11.6, red: 10.3 (%)	[20]

**Table 8:** Phenolic and vitamin C content of organic and conventional fresh produce.

Fresh Produce	Phenolic Content		References
	Organic	Conventional	
Blueberries	Bluecrop: 319.3 (mg/100 g FW)	Bluecrop: 190 (mg/100 FW)	[43]
Strawberries	Earliglow: 250 and Allstar: 145 (mg/100 g FW)	Earliglow: 190 and Allstar: 130 (mg/100 g FW)	[44]
Bell peppers	Roberta: 838.2 (mg/kg DW)	Spartacus: 833.0 and Berceo: 845.4 (mg/kg DW)	[45]
Tomatoes	Redondo: 196 (mg Gallic Acid Equivalent /100 g)	Redondo: 149 (mg Gallic Acid Equivalent /100 g)	[6]
Lettuce	Caipira: 0.247–0.256 (g/kg)	Caipira: 0.328 (g/kg)	[4]
**Vitamin C content**			
Persimmons, acerola cherries, strawberries	Rama Forte (persimmons): 12.81, Olivier (acerola cherries): 4762.84, and Oso Grande (strawberries): 55.07 (mg/100 g)	Rama Forte (persimmons): 19.49, Olivier (acerola cherries): 2294.53, Oso Grande (strawberries): 64.65 (µg/100 g)	[49]
Bell peppers	Roberta: 18.9 (g/kg)	Spartacus: 19.3 and Berceo: 18.1 (g/kg)	[45]
Strawberries	Selva: 86.4 (mg/100 g FW)	Selva: 71.2 (mg/100 g FW)	[41]
Beetroots	Libero: 207.4–209.1 (mg/kg FW)	Libero: 153.6–160.1 (mg/kg FW)	[3]
Lettuce	Caipira: 0.122–0.188 (g/kg)	Caipira: 0.096 (g/kg)	[4]

**Table 9:** Anthocyanin and β-carotene content of organic and conventional fresh produce.

Fresh Produces	β-Carotene Content		References
	Organic	Conventional	
Persimmons, acerola cherries, strawberries	Rama Forte (persimmons): 703.24, Olivier (acerola cherries): 2486.38, and Oso Grande (strawberries): 54.08 (µg/100 g)	Rama Forte (persimmons): 645.60, Olivier (acerola cherries): 6130.24, Oso Grande (strawberries): 53.02 (µg/100 g)	[69]
Tomatoes	Merkury, Akord, Rumba: 3.01 (mg)Picolino and Conchita: 5.62 (mg)	Merkury, Akord, Rumba: 2.84 (mg)Picolino and Conchita: 6.49 (mg)	[29]
Broccoli	Maine: 13.25 (fall), 29.71 (spring)Oregon: 30.10 (fall), 25.80 (spring)	Maine: 12.98 (fall), 28.73 (spring) Oregon: 29.10 (fall), 25.16 (spring)	[70]
Passion fruit	Degener: 0.06 (mg/100 g)	Degener: 0.08 (mg/100 g)	[71]
Cabbages	Sufama F1: 0.40 (mg/100 g FW)	Sufama F1: 0.37 (mg/100 g FW)	[51]
**Anthocyanin content**			
Strawberries	Diamante, Lanai, and San Juan: 205 (mg P-3-Glc equivalents/g FW)	Diamante, Lanai, and San Juan: 192 (mg P-3-Glc equivalents/g FW)	[25]
Blueberries	Powder blue: 152, Climax: 197, Tifblue: 116 (mg/100 g FW)	Powder blue: 140, Climax: 224, Woodward: 183 (mg/100 g FW)	[72]
Strawberries	Selva: 19.3 (cyanidin-3-glucoside) and 332.3 (pelargonidin-3-glucoside) (µg/g FW)	Selva: 9.8 (cyanidin-3-glucoside) and 254.1 (pelargonidin-3-glucoside) (µg/g FW)	[41]

**Table 10:** Flavonoid and lycopene content of organic and conventional fresh produce.

Fresh Produce	Flavonoid Content		References
	Organic	Conventional	
Apples	Starking Delicious: 1.61 (mg CAE/g FW)	Starking Delicious: 1.88 (mg CAE/g FW)	[27]
Tomatoes	Merkury, Akord, Rumba: 61.60 (mg/100 g)Picolino and Conchita: 107.11 (mg/100 g)	Merkury, Akord, Rumba: 56.79 (mg/100 g)Picolino and Conchita: 89.96 (mg/100 g)	[29]
Beetroots	Libero: 99.3–100 (g/kg FW)	Libero: 90.71–116.5 (g/kg FW)	[3]
Cabbages	Sufama F1: 3.95 (g/100 g FW)	Sufama F1: 4.36 (g/100 g FW)	[51]
**Lycopene content**			
Persimmons	Rama Forte: 567.87 (µg/100 g)	Rama Forte: 453.27 (µg/100 g)	[69]
Tomatoes	Merkury, Akord, Rumba: 224.04 (mg)Picolino and Conchita: 111.04 (mg)	Merkury, Akord, Rumba: 211.70 (mg)Picolino and Conchita: 117.66 (mg)	[29]
Tomatoes	Redondo: 2.19 (mg/100 g)	Redondo: 1.76 (mg/100 g)	[6]
Passion fruit	Degener: 0.002 (mg/100 g)	Degener: 0.028 (mg/100 g)	[71]

**Table 11:** Antioxidant activity of organic and conventional fresh produce.

Fresh Produce	Antioxidant Activity		References
	Organic	Conventional	
Apples	Starking Delicious: 890, Golden Delicious: 242, Granny Smith: 634, Royal Gala: 477, Jona Gold: 582 (µmol Ferric Reducing Antioxidant Power; FRAP/100 g FW)	Starking Delicious: 701, Golden Delicious: 210, Granny Smith: 640, Royal Gala: 494, Jona Gold: 479 (µmol FRAP/100 g FW)	[77]
Strawberries	Diamante, Lanai, and San Juan: 11.88 (mmol Trolox equivalents/g FW)	Diamante, Lanai, and San Juan: 10.9 (mmol Trolox equivalents/g FW)	[25]
Blueberries	Powder blue: 48.9, Climax: 52.7, Tifblue: 44.7 (µmol Trolox equivalents/g FW)	Powder blue: 44.4, Climax: 55.7, Woodward: 52.6 (µmol Trolox equivalents/g FW)	[72]
Apples, pears, carrots, blackcurrants, beetroots, and celery	Red Boskoop (apples): 19,720, Bartlett (pears): 3657, Tiben (blackcurrants): 98,432, Perfection (carrots): 1298, Czerwona Kula (beetroots): 31,574, and Jabłkowy (celery): 3582 (µmol FRAP/100 g FW)	Red Boskoop (apples): 17,092, Bartlett (pears): 2796, Tiben (blackcurrants): 108,802, Perfection (carrots): 1279, Czerwona Kula (beetroots): 24,055, and Jabłkowy (celery): 2022 (µmol FRAP/100 g FW)	[28]
